# The Refraction Assessment and the Electronic Trial Frame Measurement during Standing or Sitting Position Can Affect Postural Stability

**DOI:** 10.3390/ijerph19031558

**Published:** 2022-01-29

**Authors:** Massimo Rossato, Alessandra Nart, Giuseppe Messina, Francesco Favro, Valentina Rossato, Enxhi Rrutja, Vincenzo Biancalana

**Affiliations:** 1 Department of Biomolecular Sciences, University of Urbino, 61029 Urbino, Italy; alessandra.nart@unipd.it (A.N.); vincenzo.biancalana@uniurb.it (V.B.); 2 Postural Equipe Academy, 30033 Venice, Italy; 3 Department of Biomedical Sciences, University of Padova, 35131 Padova, Italy; 4 Sport and Exercise Sciences Research Unit, Department of Psychological, Pedagogical and Educational Sciences, University of Palermo, 90128 Palermo, Italy; giuseppe.messina17@unipa.it; 5 Department of Biomedical Sciences, School of Human Movement Science, University of Padova, 35128 Padova, Italy; francesco.favro@studenti.unipd.it; 6 Department of Medicine, School of Medicine, University of Padova, 35128 Padova, Italy; valentinarossato95@gmail.com; 7 School of Science, Optics and Optometry, University of Florence, 50121 Firenze, Italy; enxhi.rrutja@adaptica.com

**Keywords:** vision, posture, postural balance, posture physiology, computerized occlusion analysis, temporomandibular disorders (TMD), refractometry, prescribing glasses

## Abstract

Vision has been shown to influence body posture. The purpose of this study is to investigate the correlations between visual acuity and body postural control both in a standing and seated position. This cohort study included 37 patients examined using Adaptica’s (Italy) Kaleidos and VisionFit. Objective refraction was measured with Kaleidos both in a standing and seated position by the same operator and in the same environmental conditions. The parameters obtained with the device were binocular refraction, monocular refraction, pupil distance, pupil size, head tilt, gaze, phorias, and tropias. The results obtained were then subjectively tested using VisionFit: an electronic trial frame with phoropter functionalities. The study’s outcome revealed that the differences in the visual acuity parameters obtained in standing and seated positions were statistically significant; the Student’s *t*-test showed a *p*-value < 0.001 in all parameter averages. Automated refraction is widely being performed and postural control can affect the visual acuity parameters; therefore, it is relevant to consider the possibility of measuring in orthostatism. It might be appropriate to take into account the possibility of measuring in orthostatism and wearing trial frames in orthostatic conditions as well as walking freely around the room, looking outside of a window, sitting, and reading.

## 1. Introduction

Postural control is a summation of postural reflexes [1]. Postural body schema is influenced by head position [2], adapting to the head movements regulated by sensory feedback from at least the vestibular system, vision, oculomotor muscles, and dental occlusion [3,4,5,6,7,8].

Refractive errors have been correlated with interfered postural control and modified dental occlusion [9]. The clinical correlation between the stomatognathic system and the body is still much debated in the literature and conflicting data has been reported [3,10,11].

The objective determination of refraction is necessary for subjective adjustment aimed at prescribing glasses [12,13]. Nevertheless, uncorrected refractive error is the leading cause of visual impairment and has significant implications in the patient’s quality and economy of life [14,15]. The primary sensory information to maintain postural balance is given by the visual system [16,17,18]. Posture is controlled by the postural control (PC) or fine postural system (SPF) through a complex network of synaptic pathways that connect the periphery to the subcortical nuclei (ganglia), the cerebellum, and the cortex [17,19]. The vestibular system is sensitive and specializes in working in very low frequencies such as those of head movements [20]. The system for otoliths, sacculum, and utricle specializes in linear accelerations and decelerations, angular type, and head rotations in the three planes of space according to the orientation of the three right and left semi-circular channels (with small inclinations of a few degrees), working in pairs on both sides. This large amount of redundant information has never been lost by our species during the course of its evolution, proof of its efficacy and necessity [21]. Through static stabilometry, with the patients standing, first with their eyes open and then with their eyes closed, Toupet (1991) showed that viral vestibular neuritis does not cause the CoP to move towards the pathological side, but there was only an increase in the ellipse sway area of kinesigram [22].

Jethani (2017) demonstrated the presence of a “new anatomical landmark” in the form of a ridge-like structure at the insertion site of the medial rectus muscle, located at a distance of approximately two-thirds from the superior part of the medial rectus insertion; this ridge may be of functional importance in ocular motility [23]. Thus, contrary to its name, the medial rectus muscle is not as “straight” as the Latin rectus would suggest, something that becomes a relevant factor in understanding why and how the eye is key in relation to learning disabilities, postural correction, and sports training. The medial rectus muscle is found to be shorter than the lateral rectus muscle, having shorter tendons and being attached closer to the limb. Furthermore, compared to the lateral rectus muscle, it has a greater insertion base, a different ratio of elastin to smooth fibers, and a mobile functional pulley [23,24]. To posturologists, the importance of the medial rectus muscle is found in the fact of its connection to the vestibular system with which it controls some of the main postural muscles, from the trapezius to the sternocleidomastoid and to the soleus [25]. Together with vision muscles, the medial rectus muscle is necessary for ancestral decision making, such as whether to attack or escape, and together with the vestibular system, it is necessary to keep in binocular foveal vision prey or predators, allowing to experience visual flux and movement scrolling [26,27,28]. In a reductive way, the proprioceptive system receives information from the proprioceptive receptors and integrates it with all the information it receives from all the other sensory receptors [29]. Good posture is investigated standing up, straight, and still, with the arms hanging naturally to the sides. Then, we evaluate the misalignment of the musculoskeletal system with the feet open at the distance of the hip width [26,27,30]. Roll (1987) demonstrated, through the vibration of the oculomotor muscles (approximately 30% of the optic nerve fibers), that the body in an upright position falls on the opposite side to that stimulated [27]. Then, Roll (1997) demonstrated the orientative function of the upper part of the body (main receptor for the eyes) and the adaptive and compensatory function of the lower part (feet) only in an upright condition [31]. Kapoula (2006) investigated and demonstrated that postural stability is related to the functionality of the visual system by testing the effect of gaze position and convergence (experimented with prisms) in 18 young and 17 elderly subjects in standing position [32,33]. Rossato (2020) published in the book, *Posturology 4.0*, how all postural sensory afferents in standing position are to be tested by means of stabilometry [27,30]. Hansson (2010) demonstrated the presence of postural control integrations between the visual and vestibular synaptic afferents by measuring standing [34]. Schärli (2012) demonstrated the influence of gaze behavior on postural control from early childhood into adulthood in quiet standing on a stabilometry to measure CoP displacement [35]. M. Bucci (2014) demonstrated the relation of postural control on a stable/unstable platform in three different positions (i.e., eyes open and fixed on a target, eyes under optokinetic stimulation, and eyes closed) by spatial and temporal analysis of CoP in 46 children, standing [36,37].

The conclusion that emerges is that a correlation between vision (approximately 70% of the optic nerve fibers) and postural control is demonstrated. Postural control is shown to be measured and objectified in an orthostatic position.

The starting point for this research was the simple consideration: Why measure visual acuity in a seated position when posture is assessed in orthostatism and the eye (with the oculomotor system) generates postural compensation in an erect position? From this is logically derived the question: Why are trial lenses used in a seated position? [38,39].

The hypothesis of this study was the need of an orthostatic position for an objective evaluation of vision and trial lenses. 

After a search on MEDLINE easily showed there were few scientific works offering an explanation, we ultimately arrived at the study of the effect of vision on sit-to-stand movement [40,41] and at the study of which the sensory system predominates in maintaining postural balance [27,41,42,43].

## 2. Materials and Methods

**Figure 1:**
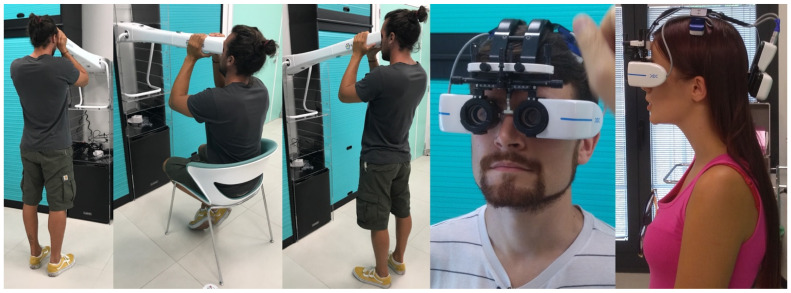
The different measurement conditions using Kaleidos in standing and seated position. The last 2 photos show measurement using VisionFit SC, an electronic wearable adaptive refractor.

The measurements were performed using Adaptica’s (Padua, Italy) mobile devices: Kaleidos and VisionFit. Kaleidos is a binocular mobile refractometer and vision analyzer that measures binocular refraction and discovers other ocular impairments. The device serves as a darkroom, allowing for the examination to be performed under any light conditions; while the patient looks inside of it, the system automatically detects refractive errors in less than three seconds. The measuring principle of the device is based on eccentric photo-retinoscopy. The device accepts a pupil diameter between 3.5 and 11 mm. This device performs measurement during a continuous tracking of the corneal reflex for the analysis of the binocular alignment of refractive errors: myopia and hyperopia in the range of ±15 D. The cylinder axis is calculated between 1° and 180° (step 1°). This device performs the “reliability index”, a number between R1 and R9 (the higher the index, the more reliable the measure). During the binocular measurement, it displays the pupils’ diameters and the horizontal and vertical offset angles of the average position of the corneal reflex in respect to the center of the detected pupil, the pupillary distance, and the inclination of the line connecting the centers of the two pupils. Axis angles were expressed according to the TABO system. The Kaleidos refractometer (Figure 1) was investigated in a scientific work that showed good correlation to the outcomes of the Retinomax K-plus2 and NIDEK RKT-7700 Autorefractor [44,45], considered gold standards in the field.

VisionFit SC is an electronic, mobile, and wearable adaptive refractor added reference (Figure 1). It replaces both the trial frame and the manual or electronic phoropter functionalities; the trial lenses’ ranges are provided by a liquid lens, automatically and easily controlled through a tablet, allowing for the adjustment of power diopters, axis, and JCC; the peripheral vision is wider than any other phoropter available. Subjective refraction can be performed with the patient in any position and location due to the mobility and wearability of the unit. This allows the patient to move freely around the room enabling accurately measurement in a more comfortable way.

The cohort was obtained from a total of 38 healthy volunteers recruited on Facebook of which 18 were male and 21 were female from 18 to 32 years of age.

The exclusion criteria were a previous diagnosis of ametropia, eye pathologies, recent eye surgery, or recent traumatic injury to the body or lower limbs. One of the volunteers was excluded as he suffered from Cogan’s syndrome; as a consequence, the total number of accepted subjects was 37 with an average age of 25.7 ± 3.5.

The research was conducted in Rossato’s clinic (A.T.I.P.—Padua, Italy) as follows: Every patient underwent objective refraction with Kaleidos in orthostatism and, after a few minutes, in a sitting position. The two results were then imported into the VisionFit’s control unit in order to proceed with the examination. Each patient, standing at a three meter distance from an optotype, read the letters with their own glasses up to 10/10 acuity in order to have a starting point for the quality of their vision. The subjects then wore the VisionFit and repeated the chart reading in orthostatism with both the objective measurements quickly interchanged through the device’s control unit. Afterwards, the patients were asked a subjective opinion on the two configurations when switching from one to the other through the following questions: “Is it better or worse than your glasses?” “Better now (configuration 1) or like this (configuration 2)?” “Read the last line, do you see it better now (configuration 1) or like this (configuration 2)?”. All subjects gave their informed consent for inclusion before they participated in the study and written informed consent was obtained from participating patients. This work was known to the University of Urbino, and the methods, material, and study design employed were observed from its inception. In light of this, it was decided that it did not require the authorization of the Institutional Review Board or an ethical approval statement.

This clinical trial was in line with the 2008 version of the World Medical Association’s Declaration of Helsinki and good clinical practice.

The subjects were not informed of which kind of refractive measure they were being subjected to in order to avoid bias. They were simply asked to express preference based on their best visual perception.

All subjects were measured by the same operator and in the same environmental conditions, both in an orthostatic position and in a seated position.

During the overall postural assessment of the patient, we always conducted measurement of the ocular laterality, because it is correlated with the activity of the oculomotor muscles (30% of the afferents of the optic nerve). During this study we focused on vision (70% of the optic nerve afferents) and, therefore, we did not report laterality.

Statistical analysis was performed on the objective refraction outcomes, verifying the presence and significance of differences between the two measurements.

The Student’s *t*-test for paired samples was performed with a confidence interval of 95% and significance at *p* < 0.05.

The patient’s lifestyle was also investigated, checking whether they had an active, sedentary, or athletic life, because there may be a relationship with the standing position [46,47].

Afterwards, the χ2 test was performed on the qualitative data of objective refraction to verify how much the observed frequencies deviated from the expected ones (with a tolerated error of 5%).

## 3. Results

In Table 1, the main statistical center and dispersion indices for the database are depicted, separated into the two configurations (i.e., standing person/sitting person). Data regarding the axes of astigmatism were included for completeness, but indices such as maximum and minimum have little meaning for these periodic variables. We compared the distribution of the individual parameters to the distribution present in the literature [45]. 

**Figure 2:**
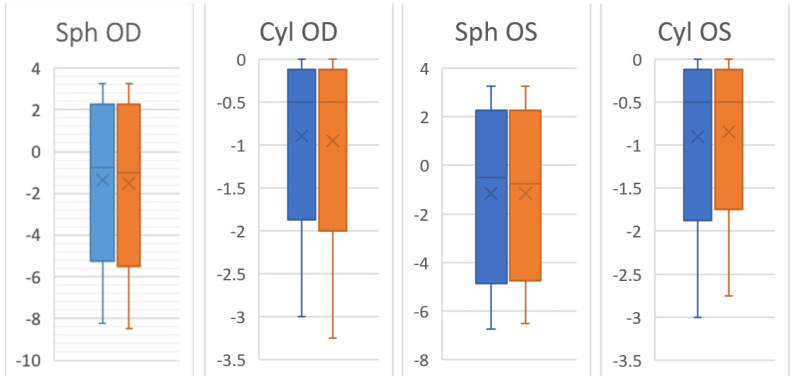
Dispersions statistics in the standing Position (blue) and the sitting position (orange). Sph OD = right eye sphere; Cyl OD = right eye cylinder; Sph OS = left eye sphere; Cyl OS = left eye cylinder.

The maximum value for astigmatism is always 0, since it corresponds to the condition of a non-astigmatic eye. It can be noted that the data relating to “seated” measurements were slightly more dispersed for the right eye but less dispersed for the left eye. This is also confirmed by Figure 2.

All average differences were different from zero, which proves that the hypothesis that there is a difference between the seated and standing positions was true. Finally, all values showed a significant statistical difference, far behind the 95% confidence. 

Table 2 shows the absolute differences between the two data sets (i.e., sitting and standing) obtained with the subtraction between members and applying the absolute value to the result.

In Table 2, the axes of astigmatism were corrected to consider their periodicity. For example, the difference in patient 23 for the left eye would be 0 − 177° = −177°, but the actual difference was of only 3°.

In Table 3 are reported the main indices of center and dispersion related to the differences—in absolute value—between the two data sets as presented in Table 2. 

In Table 4, the Student’s *t*-test confirmed the statistical significance (*p* < 0.05) of the results with the following values associated with the respective *p*-value.

The Student’s *t*-test showed a *p* < 0.001 for all averages.

In Table 5 is reported the lifestyle conducted by the patients.

**Figure 3:**
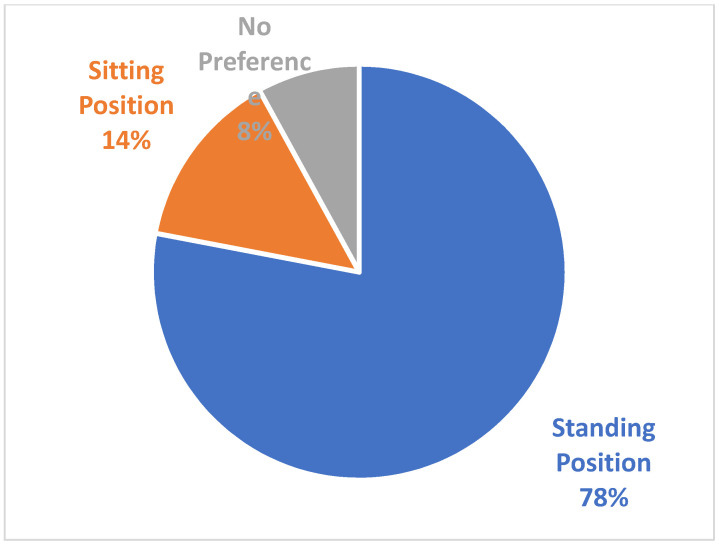
Patients’ subjective preference of the trial lenses.

The patient’s subjective preference of the two refractive configurations obtained during the sitting or standing position is shown in Figure 3.

It appears evident how a strong majority of the population preferred the measurement obtained in the standing position. The χ2 test with 2 degrees of freedom gave the following results: 

χ2 = 35.86, *p* = 0.00000002.

## 4. Discussion

The aim of this study was to verify if the refractometer vision measurements and an objective evaluation of the trial lenses had a difference statistically significative between the orthostatic and sitting position. 

Table 3 shows that for at least 25% of the subjects in each category (except Ax LE), the difference between the “standing” and “sitting” measures remained zero, while for at least 50% of the subjects, the difference between the two measurements was equal to or greater than 0.25 D (except Cyl RE).

Cyl RE presented as the least dispersed data. At least half of the subjects had no difference between the measures, even if we cannot advance a hypothesis on the causes of this lower dispersion.

Six subjects found a non-zero difference in each category. One subject found a difference of at least 0.5 D in each category and differences greater than Q3 in the axes of astigmatism; to the subjective refraction, he immediately indicated the standing measurement as the best.

The maximum observed difference was 0.75 D in all categories (except Cyl RE, where maximum |D| was 0.5 D). The maximum observed difference in the axes of astigmatism was 58% higher in the left eye compared to the right one, which has less dispersion. Similarly, Sph and Cyl were less dispersed for the right eye than for the left. It can be hypothesized that this different dispersion is linked to the prevailing dominance of the right eye [48], considering the relationship that seems to exist between ocular dominance and posture.

The responses obtained from the subjective refraction, performed in the standing position, agreed with the objective data: 34 out of 37 subjects found a perceptible difference between the two configurations transferred into the VisionFit.

Three subjects reported that they did not perceive differences between the two configurations in accordance with the objective data. In fact, two out of three, had no differences between the two measures.

No relevant trend emerged in subjects who preferred the “seated” configuration.

The fact that most participants preferred the “standing” measurement can be attributed to the subjective choice being performed in an upright position. Future research could compare the results of subjective refraction taken both in standing and sitting positions and verify if the subjects express a preference for one of the two configurations. 

In a 2001 study, Atchison et al. [49] found that a difference of 0.25 D compared to a pair of glasses deemed correct for the person was already sufficient to make subjects perceive an “uncomfortable” vision, while almost all of the samples found the incorrect 0.5 D lens “unacceptable”. Considering this information, the difference of at least 0.25 reported in approximately half of the cases appears even more significant.

In the context of this study, only static visual acuity was evaluated. Future research lines could explore whether there are differences when the analysis is conducted in a sitting or standing position also for other visual abilities and skills (dynamic visual acuity, peripheral vision, etc.). 

## 5. Conclusions

There was a correlation between postural control and visual functions as the mandibular position and the vestibular apparatus. Automated refractometry is widely performed. As the statistical data analysis revealed, the visual acuity parameters seemed to point to a strong correlation with body position.

The overall postural control will therefore be assumed to differ in the orthostatic position from the sitting one.

It might be appropriate to take into account the possibility of measuring in orthostatism and wearing trial frames in orthostatic conditions as well as walking freely around the room, looking outside of a window, sitting, and reading.

## Figures and Tables

**Table 1:** Statistics in the standing and sitting positions. RE = right eye; LE = left eye; Sph = sphere; Cyl = cylinder; Ax = axis; SD = standard deviation; Q1 = first quartile (25%); Q3 = third quartile.

	Standing Position						Sitting Position					
	RE			LE			RE			LE		
	Sph	Cyl	Ax	Sph	Cyl	Ax	Sph	Cyl	Ax	Sph	Cyl	Ax
Average	−0.99	−0.68	91.46	−1.01	−0.62	73.39	−0.98	−0.68	91.19	−0.93	−0.67	85.16
SD	2.56	0.61	70.08	2.39	0.63	73.18	2.52	0.64	72.06	2.36	0.62	72.06
Minimum	−8.25	−3	0	−6.75	−3	0	−8.5	−3.25	0	−6.5	−2.75	0
Q1 (25°p)	−2.25	−0.75	20	−3	−0.75	4	−2.5	−0.75	9	−3	−0.75	11
Median	−0.75	−0.5	102	−0.5	−0.5	41	−1	−0.5	98	−0.75	−0.5	57
Q3 (75°p)	1.25	−0.25	158	1.25	−0.25	159	1.25	−0.25	161	1.25	−0.25	163
Maximum	3.25	0	176	3.25	0	179	3.25	0	176	3.25	0	180

**Table 2:** Absolute differences between the standing and sitting positions. RE = right eye; LE = left eye; Sph = sphere; Cyl = cylinder; Ax = axis.

	RE			LE		
Patients	Sph	Cyl	Ax	Sph	Cyl	Ax
1	0.25	0.25	1	0	0.5	8
2	0	0	0	0	0	0
3	0	0.25	2	0	0	5
4	0.25	0	5	0.25	0.25	5
5	0.25	0.25	9	0.25	0.25	0
6	0	0.25	0	0.75	0.5	2
7	0.5	0.25	3	0	0.25	0
8	0.25	0	0	0	0	14
9	0.25	0	3	0	0.25	3
10	0	0	1	0	0.25	12
11	0.75	0.5	24	0.5	0.75	21
12	0	0	0	0	0	12
13	0.5	0	7	0.25	0	12
14	0.25	0.25	3	0.25	0.25	2
15	0.5	0.25	0	0	0	6
16	0	0	7	0.25	0.25	17
17	0.25	0,5	12	0.25	0	13
18	0	0	17	0.25	0.25	4
19	0	0	5	0.25	0.25	38
20	0.25	0.25	10	0.25	0.25	3
21	0	0.25	6	0.25	0	19
22	0	0	5	0	0.25	3
23	0.25	0	0	0.5	0.5	36
24	0.25	0	10	0.25	0	0
25	0.25	0	3	0	0.25	10
26	0	0.5	6	0.75	0.5	4
27	0.25	0.25	5	0.25	0.25	3
28	0.25	0	14	0	0.25	14
29	0	0	0	0	0.5	10
30	0	0.25	0	0	0	1
31	0.25	0.25	2	0.5	0.25	2
32	0.25	0	0	0	0	6
33	0	0	6	0.25	0.25	3
34	0.25	0	10	0	0	0
35	0.5	0.25	2	0	0	3
36	0.25	0	0	0	0.25	23
37	0	0	0	0.25	0.25	4

**Table 3:** Center and dispersion of absolute value between the standing and sitting positions. RE = right eye; LE = left eye; Sph = sphere; Cyl = cylinder; Ax = axis; SD = standard deviation; Q1 = first quartile (25%); Q3 = third quartile.

	RE			LE		
	Sph	Cyl	Ax	Sph	Cyl	Ax
Average _|D|_	0.19	0.13	4.8	0.18	0.21	8.6
SD _|D|_	0.19	0.16	5.49	0.21	0.19	9.31
Variance _|D|_	0.04	0.03	30.16	0.05	0.04	86.69
Minimum _|D|_	0	0	0	0	0	0
Q1 (25°p) _|D|_	0	0	0	0	0	3
Median _|D|_	0.25	0	3	0.25	0.25	5
Q3 (75°p) _|D|_	0.25	0.25	7	0.25	0.25	12
Maximum _|D|_	0.75	0.5	24	0.75	0.75	38

**Table 4:** Student’s *t*-test. RE = right eye; LE = left eye; Sph = sphere; Cyl = cylinder; Ax = axis.

	RE			LE		
	Sph	Cyl	Ax	Sph	Cyl	Ax
t	6.05	4.8	5.33	5.06	6.67	5.62
*p*	0.000001	0.00003	0.000005	0.00001	0.0000001	0.000002

**Table 5:** Lifestyle conducted by the patients.

Athletic Lifestyle	12	32%
Active lifestyle	20	54%
Sedentary lifestyle	5	14%

## Data Availability

Data supporting reported results are stored in accordance with the GDPR privacy practices at Rossato’s Clinic.
